# Emergence of Japanese encephalitis virus genotype V in the Republic of Korea

**DOI:** 10.1186/1743-422X-8-449

**Published:** 2011-09-23

**Authors:** Ratree Takhampunya, Heung-Chul Kim, Bousaraporn Tippayachai, Ampornpan Kengluecha, Terry A Klein, Won-Ja Lee, John Grieco, Brian P Evans

**Affiliations:** 1 Department of Entomology, United States Army Medical Component, Armed Forces, Research Institute of Medical Sciences, APO AP 96546, Bangkok, Thailand; 2 5th Medical Detachment, 168th Multifunctional Medical Battalion, 65th Medical Brigade, Unit 15247, APO AP 96205-5247, Yongsan, Seoul, Republic of Korea; 3 Force Health Protection and Preventive Medicine, 65th Medical Brigade/USAMEDDAC-Korea, Unit 15281, APO AP 96205-5281, Yongsan, Seoul, Republic of Korea; 4 National Institute of Health, Korea Centers for Disease Control and Prevention, Cheongwon gun, Chungbuk Province, 363-951, Republic of Korea; 5 Department of Preventive Medicine and Biometrics, Uniformed Services University of Health Sciences, 4301 Jones Bridge Road, Bethesda, MD 20814, USA

**Keywords:** Japanese encephalitis virus, genotype I, genotype V, *Culex tritaeniorhynchus*, *Culex bitaeniorhynchus*, Muar

## Abstract

**Background:**

Japanese encephalitis virus (JEV) genotype V reemerged in Asia (China) in 2009 after a 57-year hiatus from the continent, thereby emphasizing a need to increase regional surveillance efforts. Genotypic characterization was performed on 19 JEV-positive mosquito pools (18 pools of *Culex tritaeniorhynchus *and 1 pool of *Cx. bitaeniorhynchus*) from a total of 64 positive pools collected from geographically different locations throughout the Republic of Korea (ROK) during 2008 and 2010.

**Findings:**

Two regions of the JEV genome were sequenced from 19 pools; the envelope gene and the nonstructural protein 5 (NS5)/3'-untranslated region (UTR). Eighteen pools of *Culex tritaeniorhynchus *and one pool of *Cx. bitaeniorhynchus *were positive for genotype I and genotype V, respectively. Sequence alignment of the complete E gene from *Cx. bitaeniorhynchus *showed high amino acid similarity (98.8%) to the Muar strain, characterized as the first report of genotype V, isolated from an encephalitis patient in Malaysia in 1952.

**Conclusion:**

This study represents the first report of JEV genotype V in the ROK. The reemergence of genotype V in Asia (China and ROK) after more than a half-century and its discovery in *Cx. bitaeniorhynchus*, a mosquito species previously unknown to carry JEV in the ROK, emphasizes the need for enhanced JE surveillance to monitor the dynamics of JEV strains within the region. Future findings may have implications with regard to JEV vaccination/prevention strategies.

## Background

Japanese encephalitis virus (JEV) is a mosquito-borne member of the family *Flaviviridae*, genus *Flavivirus*, and a primary cause of viral encephalitis in humans within its range [1]. The positive-sense RNA viral genome is approximately 11 kb in length and is translated into three structural proteins [Capsid (C), Membrane (M), and Envelope (E)] and seven nonstructural (NS) proteins (NS1, NS2A, NS2B, NS3, NS4A, NS4B, and NS5) with untranslated regions (UTR) at the 5' and 3' ends of the genome [2]. Historically, *Culex tritaeniorhynchus *was implicated as the primary vector of JEV in the Republic of Korea (ROK) and much of Asia [3,4]. However, JEV has since been detected in additional culicine species throughout its range, including *Cx. bitaeniorhynchus *from the ROK [5]. JEV strains are generally classified into five genotypes (genotypes I, II, III, IV, and V) based on similarities in the E gene nucleotide sequence [6]. Previously, only genotype I was detected on the Korean peninsula [7]. Therefore, we characterized JEV-positive pools of *Cx. tritaeniorhynchus *and *Cx. bitaeniorhynchus *to determine whether the unexpected finding of JEV in *Cx. bitaeniorhynchus *in the ROK may have coincided with the appearance of an additional genotype.

## Materials and Methods

**Figure 1:**
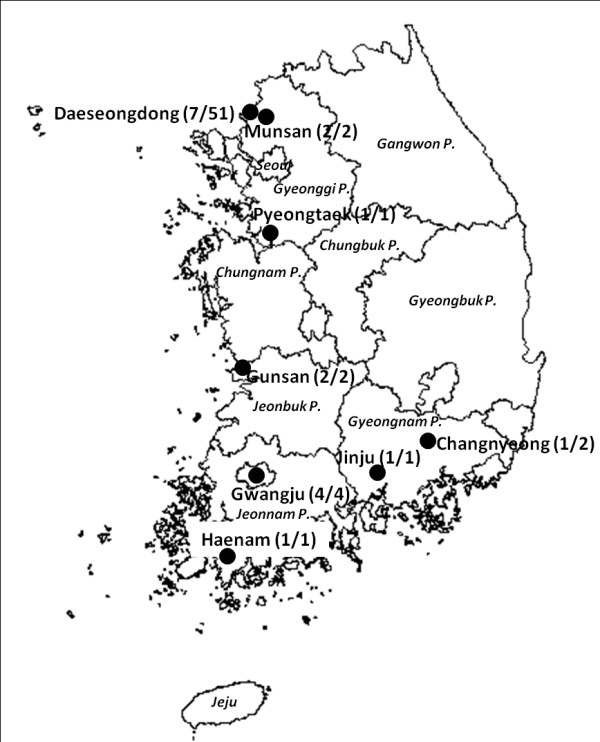
**Locations of JEV-positive mosquito pools collected during 2008 and 2010 in the Republic of Korea**. Daeseongdong is a village near the military demarcation line (MDL) (center of the 4-Km wide demilitarized zone separating North and South Korea); Warrior Base training area is approximately 5 km north of Munsan; Camp Humphreys is in a rural area of Pyeongtaek; Gunsan Air Base is located near the small city of Gunsan; Gwangju Air Base is located near the metropolitan city of Gwangju; Haenam, Jinju, and Changnyeong sites are beef/swine farms near the small cities. Pool = number of sequenced samples/total JEV-positive samples.

Nineteen JEV-positive mosquito pools, from a total of 64 JEV-positive pools collected during 2008 and 2010 in the ROK (18 pools of *Cx. tritaeniorhynchus *and 1 pool of *Cx. bitaeniorhynchus*), and one JEV culture received from USAMRIID (United States Army Medical Research Institute of Infectious Diseases, USA) were genotypically characterized (Table 1, Figure 1). Total RNA was extracted from mosquito homogenate using Trizol reagent (Invitrogen, USA) in accordance with the manufacturer's instructions and was resuspended in 50 μl of RNase-free water containing 10 units of RNasin^® ^Plus RNase Inhibitor (Promega, USA). RNA was used as the template for cDNA synthesis using the SuperScript III first strand synthesis system (Invitrogen, USA) with a random hexamer primer. The synthesized cDNA was then used for PCR amplification using iProof™ High-Fidelity DNA polymerase (Bio-Rad, USA). The NS5 gene/3' UTR and envelope (E) gene of 19 JEV-positive pools were amplified using EMF1/VD8 primers [8] and 940S/1720A primers [9], respectively. Products were purified using the QIAquick PCR purification kit (Qiagen, USA) and sequenced by AITBiotech Company (AITbiotech, Singapore).

**Table 1:** Locations and collection dates of JEV-positive mosquito pools analyzed in this study

**Collection Serial No**.	Collection Date	Collection Sites (US Military Bases, Villages/Cities)	Province	Species	**Accession no**.
A8.789	29-Jul-08	Haenam	Jeonnam	*Cx. tritaeniorhynchus*	JN587257, JN587261
A10.825	28-Sep-10	Changnyeong	Gyeongnam	*Cx. tritaeniorhynchus*	JN587255, JN587259
A10.881	21-Oct-10	Jinju	Gyeongnam	*Cx. tritaeniorhynchus*	JN587256, JN587260
10-1742	1-Sep-10	Warrior Base* (Munsan)	Gyeonggi	*Cx. tritaeniorhynchus*	JN587241
10-1748	1-Sep-10	Warrior Base* (Munsan)	Gyeonggi	*Cx. tritaeniorhynchus*	JN587242
10-1728	31-Aug-10	Daeseongdong	Gyeonggi	*Cx. tritaeniorhynchus*	JN587240
10-1937	11-Sep-10	Daeseongdong	Gyeonggi	*Cx. tritaeniorhynchus*	JN587245
10-2044	14-Sep-10	Daeseongdong	Gyeonggi	*Cx. tritaeniorhynchus*	JN587248
10-2097	21-Sep-10	Daeseongdong	Gyeonggi	*Cx. tritaeniorhynchus*	JN587249
10-2130	21-Sep-10	Daeseongdong	Gyeonggi	*Cx. tritaeniorhynchus*	JN587250
10-2357	13-Oct-10	Daeseongdong	Gyeonggi	*Cx. tritaeniorhynchus*	JN587252
**10-1827**	**8-Sep-10**	**Daeseongdong**	**Gyeonggi**	***Cx. bitaeniorhynchus***	**JN587243, JN587258**
10-1835	8-Sep-10	CP Humphreys* (Pyeongtaek)	Gyeonggi	*Cx. tritaeniorhynchus*	JN587244
10-1291	5-Aug-10	Gunsan Air Base* (Gunsan)	Jeonbuk	*Cx. tritaeniorhynchus*	JN587239
10-2204	8-Sep-10	Gunsan Air Base* (Gunsan)	Jeonbuk	*Cx. tritaeniorhynchus*	JN587251
10-1990	30-Aug-10	Gwangju Air Base* (Gwangju)	Jeonnam	*Cx. tritaeniorhynchus*	JN587246
10-1992	30-Aug-10	Gwangju Air Base* (Gwangju)	Jeonnam	*Cx. tritaeniorhynchus*	JN587247
10-2378	2-Sep-10	Gwangju Air Base* (Gwangju)	Jeonnam	*Cx. tritaeniorhynchus*	JN587253
10-2397	27-Sep-10	Gwangju Air Base* (Gwangju)	Jeonnam	*Cx. tritaeniorhynchus*	JN587254

Locations are presented in Figure 1.

* US military training site or installation.

The sequences were edited and assembled using the Sequencer program v4.1.4 (Applied Biosystems, USA). Multiple sequence alignments and phylogenetic analysis were performed using ClustalX version 2.0.11 and MEGA version 5 programs [10,11]. Percent sequence similarity/divergence was calculated using the MegAlign program found in the Lasergene v.8 software (DNASTAR, Inc., Madison, WI, USA). Phylogenetic analysis of the partial E gene (705 bp) was performed using the neighbor-joining method and Tamura-Nei model of nucleotide substitution. The maximum likelihood (ML) tree was constructed from the NS5/3'UTR nucleotide sequences (550 bp) by PhyML software v 3.0 [12] using the best fit model with aLRT branch support [13]. The ML tree for the complete E gene used the Tamura-Nei model with bootstrap analysis (2, 000 replicates) for testing the reliability of the tree using the MEGA5 (version 5) program (The Biodesign Institute, Tempe, Arizona) [11].

## Results

**Figure 2:**
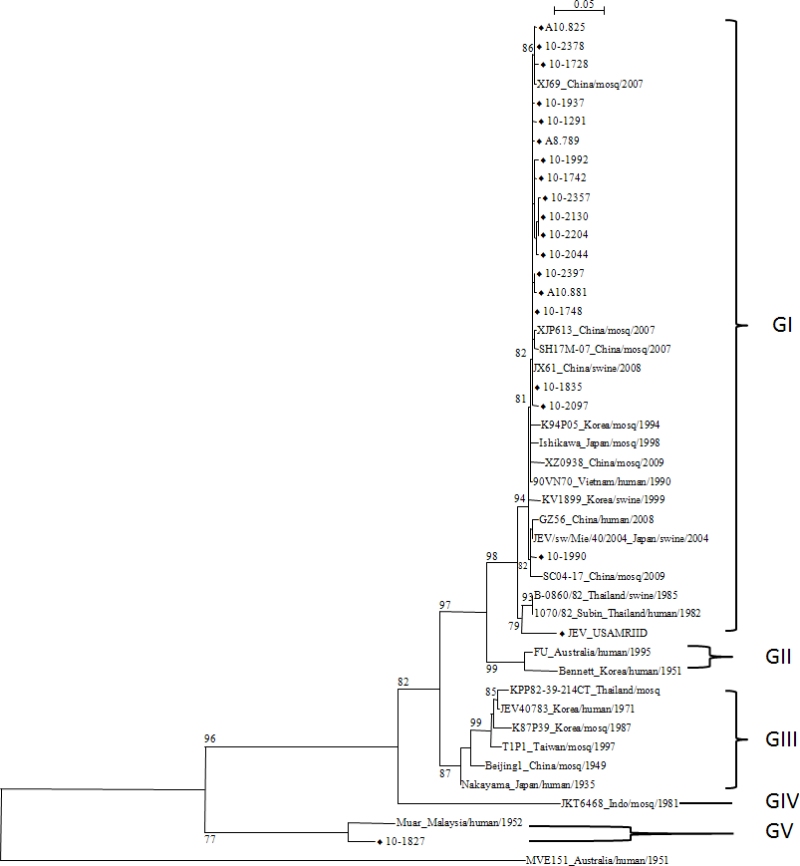
**Maximum likelihood tree of JEV strains from the ROK using NS5/3'UTR base-sequence homologies**. Phylogenetic analysis was performed using the GTR+G_4_+I model of nucleotide substitution (ln(L) = -3090.1 571) and aLRT branch support (indicated at major nodes). The phylogenetic tree has been rooted at its midpoint. Scale bar represents substitutions per site.

The phylogenetic relationships among 19 JEV strains and JEV sequences retrieved from GenBank representing genotypes I-V were analyzed. The ML tree for the NS5/3'UTR (550 bp) and the neighbor-joining tree for the partial E gene (705 bp) showed similar branching patterns with high bootstrap support. Therefore, the ML tree is only presented in this report (Figure 2). Two genotypes were identified among the 19 JEV strains. JEV strains from 18 *Cx. tritaeniorhynchus *mosquitoes grouped into genotype I. These genotype I strains were closely-related to strains isolated from China, Korea, Japan, Vietnam, and Thailand from the early 1980s to the present (Figure 2). The remaining strain from *Cx. bitaeniorhynchus *(10-1827) grouped into genotype V together with the Muar strain which was isolated from an encephalitis patient in Malaysia in 1952.

**Figure 3:**
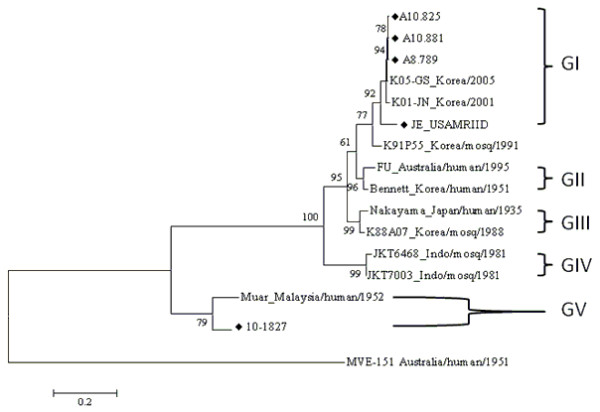
**Maximum likelihood tree of JEV strains from the ROK using complete E gene nucleotide sequence homologies**. Phylogenetic analysis was performed using the MEGA5 program by the Tamura-Nei model of nucleotide substitution (ln(L) = -7791.2). Murray Valley encephalitis virus strain (MVE-1-51) was used as an outgroup. Bootstrap values at each major node were calculated using 2, 000 replicates. Scale bar represents the number of nucleotide substitutions per site.

The complete E gene was sequenced from a subset of strains in genotype I (A10.825, A10.881, A8.789) and genotype V (10-1827). The ML tree constructed from the complete E gene of these strains together with representative JEV genotype I-V sequences is shown in Figure 3. This ML tree supports the phylogenetic analysis results performed on the NS5/3'UTR (Figure 2) and the partial E gene previously mentioned. The ML tree in Figure 3 shows that the 10-1827 strain grouped with the Muar strain with 79% bootstrap support, while the remaining sequences clustered in genotype I together with K01-JN and K05-GS strains that were isolated from *Cx. tritaeniorhynchus *in the ROK in 2001 and 2005, respectively.

Sequence analysis of 18 strains shows minimal sequence variation among viruses in genotype I, with nucleotide sequence similarity of 97.5-100% for the NS5/3'UTR (Figure 2) and 99.6-100% for the E gene (Figure 3). In an earlier study, genetic stability was also observed among JEV strains isolated from mosquito vectors in the ROK between 1994 and 2005 [7]. Examination of the complete E sequence of 10-1827 strain (genotype V) showed less similarity to the other genotypes, with nucleotide similarity approximately 77.3% (91.3% for amino acids) to genotype I (K01-JN, K05-GS), 78.1% (91.0% for amino acids) to genotype II (FU strain), 77.7% (90.4% for amino acids) to genotype III (Nakayama), and 77.8% (91.0% for amino acids) to genotype IV (JKT6468) (Table 2). However, nucleotide and amino acid similarities to the Muar strain were 90.0% and 98.8%, respectively (Table 2). Likewise, the XZ0934 strain, a JEV genotype V recently isolated from China (2009), showed E gene nucleotide and amino acid sequence similarities to the Muar strain of 86.0% and 93.2%, respectively [14].

**Table 2:** Nucleotide sequence similarity and divergence of the complete E gene from ROK mosquito pools

	A10.825	A10.881	A8.789	JE_USAMRIID	K01-JN	K05-GS	FU	Nakayama	JKT6468	10-1827	Muar
**A10.825 (1)**		99.4	99.3	94.3	98.2	99.3	89.0	87.8	81.8	77.2	76.8
**A10.881 (1)**	0.6		99.3	94.1	98.1	99.2	89.0	88.0	82.2	77.4	76.5
**A8.789 (1)**	0.7	0.7		94.3	98.2	99.3	89.1	87.8	82.1	77.2	76.4
**JE_USAMRIID (1)**	6.0	6.3	6.0		94.1	94.3	88.0	86.6	82.1	77.2	76.6
**K01-JN (1)**	1.8	1.9	1.8	6.2		98.3	89.1	87.6	81.8	77.3	76.8
**K05-GS (1)**	0.7	0.8	0.7	6.0	1.8		89.4	88.0	81.8	77.4	76.6
**FU (2)**	12.2	12.2	12.1	13.6	12.2	11.7		88.1	81.9	78.1	77.0
**Nakayama (3)**	13.8	13.5	13.7	15.3	14.1	13.6	13.3		83.0	77.7	77.5
**JKT6468 (4)**	21.8	21.1	21.3	21.3	21.7	21.7	21.5	20.1		77.8	77.1
**10-1827 (5)**	27.6	27.4	27.7	27.6	27.5	27.3	26.3	26.9	26.8		90.0
**Muar (5)**	28.2	28.6	28.7	28.4	28.1	28.5	27.9	27.2	27.7	11.1	

The upper triangle represents similarity while the lower triangle represents divergence. A8.789, A10.825, A10.881, and 10-1827 represent the ROK mosquito pools. JEV reference strains are shown for 5 genotypes: I (K01-JN, K05-GS), II (FU), III (Nakayama), IV (JKT6468), and V (Muar). Percent similarity/divergence was computed using the MegAlign program (Lasergene v.8 software, USA). Numbers in parentheses represent the JEV genotype.

**Figure 4:**
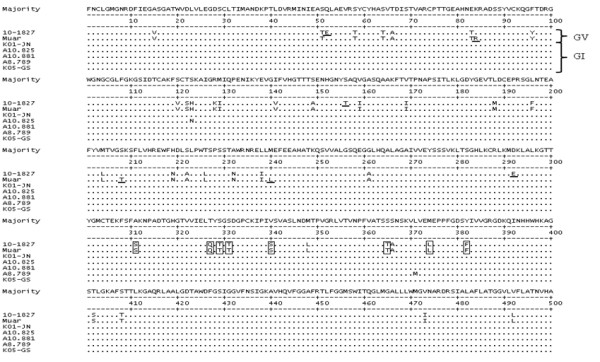
**Amino acid sequence alignment of the full-length envelope gene from ROK JEV strains**. ROK mosquito pools collected during 2008 and 2010 were aligned with the reference sequences GV (Muar) and GI (K01-JN, K05-GS). Dots indicate consensus. Differences in amino acids between Muar and 10-1827 strains are underlined. Residues enclosed by boxes represent 8 Muar signature amino acids in domain III.

Figure 4 shows the amino acid sequence alignment of the complete E gene derived from strains A10.825, A10.881, A8.789, and 10-1827 and reference sequences (Muar, K01-JN, K05-GS). The E protein of the strains in genotype I is very conserved with few amino acid changes detected: A10.825 (from S = serine to N = asparagine at position 123) and A8.789 (from L = leucine to M = methionine at position 371). The alignment reveals differences in 6 amino acid residues between the Muar and 10-1827 strains (Figure 4). The eight Muar signature amino acid residues in domain III comprising a putative receptor binding region [15] were also identified in the 10-1827 strain along with the critical amino acid residue thought to be involved in receptor binding activity (Q = glutamine at position 327) [16]. Table 3 provides a complete listing of the strains that are referenced in this study.

**Table 3:** Origin of 30 JEV strains referenced in this study

Strain	Location	Year	Host	Genotype	**Accession no**.
1070/82_Subin	Thailand	1982	Human	1	GQ902059
90VN70	Vietnam	1990	Human	1	HM228921
B-0860/82	Thailand	1985	Swine	1	GQ902058
Beijing-1	China	1949	Mosquito	3	L48961, FJ872376
Bennett	Korea	before 1951	Human	2	FJ515927
FU	Australia	1995	Human	2	AF217620
GZ56	China	2008	Human	1	HM366552
Ishikawa	Japan	1998	Mosquito	1	AB051292
JEV/sw/Mie/40/2004	Japan	2004	Swine	1	AB241118
JEV40783	Korea	before 1971	Human	3	FJ515923
JKT6468	Indonesia	1981	Mosquito	4	AY184212
JKT7003	Indonesia	1981	Mosquito	4	U70408
JX61	China	2008	Swine	1	GU556217
K01-JN	Korea	2001	Mosquito	1	FJ938222
K87P39	Korea	1987	Mosquito	3	AY585242
K88A07	Korea	1988	Mosquito	3	FJ938227
K91P55	Korea	1991	Mosquito	-	U34928
K94P05	Korea	1994	Mosquito	1	AF045551
KO5-GS	Korea	2005	Mosquito	1	FJ938223
KPP82-39-214CT	Thailand	-	Mosquito	3	GQ902063
KV1899	Korea	1999	Swine	1	AY316157
Muar	Malaysia	1952	Human	5	HM596272
MVE-1-51	Australia	1951	Human	-	AF161266
Nakayama	Japan	1935	Human	3	EF571853
SC04-17	China	2004	Mosquito	1	GU187972
SH17M	China	2007	Mosquito	1	EU429297
T1P1	Taiwan	1997	Mosquito	3	AF254453
XJ69	China	2007	Mosquito	1	EU880214
XJP613	China	2007	Mosquito	1	Eu693899
XZ0938	China	2009	Mosquito	1	HQ652538

All strains are JEV, with the exception of MVE-1-51, a strain of Murray Valley encephalitis virus.

## Conclusion

This study is the first report of JEV genotype V in the ROK and represents the third report of genotype V in Asia, with the most recent findings from *Cx. tritaeniorhynchus *collected in Tibet, China (2009) [14]. The fact that JEV genotype V, first reported from an encephalitis patient in Malaysia in 1952 (Muar strain), came long before the discovery of its reemergence in China in 2009 and now its subsequent appearance in the ROK may mark the beginning of a genotypic shift in JEV within the region. Additionally, the emergence of this strain in *Cx. bitaeniorhynchus*, a mosquito species previously unknown to carry JEV in the ROK, underscores the need to step-up surveillance efforts within the ROK. The reemergence of this genotype after 57 years may have future implications with regard to JEV vaccination effectiveness and policy among civilian and military populations, as well as with preventive strategies designed to reduce the health impact and incidence of JEV among at risk Asian populations.

## Competing interests

The authors declare that they have no competing interests.

## Authors' contributions

RT and BPE conceived the study, the design, and drafted the manuscript. RT, BT, and AK carried out all molecular work. HCK, WJL, and TAK collected the mosquitoes and assisted in drafting the manuscript. JG appropriated funding (program protocols) and participated in conducting the study. All authors read and approved the final manuscript.

## References

[B1] Tsai TF New initiatives for the control of Japanese encephalitis by vaccination: minutes of a WHO/CVI meeting, Bangkok, Thailand, 13-15 October 1998 Vaccine 2000 18 Suppl 2 1 25 10821969 10.1016/s0264-410x(00)00037-2

[B2] Lindenbach BD Rice CM Flaviviridae: The Viruses and Their Replication Lippincott Williams & Wilkins, Philadelphia, PA 2001 421866005

[B3] Scherer WF Buescher EL Flemings MB Noguchi A Scanlon J Ecologic studies of Japanese encephalitis virus in Japan. III. Mosquito factors. Zootropism and vertical flight of *Culex tritaeniorhynchus *with observations on variations in collections from animal-baited traps in different habitats Am J Trop Med Hyg 1959 8 665 677 14442650

[B4] Solomon T Control of Japanese encephalitis--within our grasp? N Engl J Med 2006 355 869 871 10.1056/NEJMp058263 16943399

[B5] Kim HC Terry KA Takhampunya R Evans BP Mingmongkolchai S Kengluecha A Grieco J Masuoka P Kim MS Chong ST Lee JK Lee WJ Japanese encephalitis virus in culicine mosquitoes (Diptera: Culicidae) collected at Daeseongdong, a village in the Demilitarized zone of the Republic of Korea J Med Entomol 2011 in press 10.1603/me11091 22238887

[B6] Solomon T Ni H Beasley DW Ekkelenkamp M Cardosa MJ Barrett AD Origin and evolution of Japanese encephalitis virus in southeast Asia J Virol 2003 77 3091 3098 10.1128/JVI.77.5.3091-3098.2003 12584335 PMC149749

[B7] Yun SM Cho JE Ju YR Kim SY Ryou J Han MG Choi WY Jeong YE Molecular epidemiology of Japanese encephalitis virus circulating in South Korea, 1983-2005 Virol J 2010 7 127 10.1186/1743-422X-7-127 20546562 PMC2893154

[B8] Pierre V Drouet MT Deubel V Identification of mosquito-borne flavivirus sequences using universal primers and reverse transcription/polymerase chain reaction Res Virol 1994 145 93 104 7520190 10.1016/s0923-2516(07)80011-2

[B9] Schuh AJ Li L Tesh RB Innis BL Barrett AD Genetic characterization of early isolates of Japanese encephalitis virus: genotype II has been circulating since at least 1951 J Gen Virol 2010 91 95 102 10.1099/vir.0.013631-0 19776238 PMC2885061

[B10] Thompson JD Gibson TJ Plewniak F Jeanmougin F Higgins DG The CLUSTAL_X windows interface: flexible strategies for multiple sequence alignment aided by quality analysis tools Nucleic Acids Res 1997 25 4876 4882 10.1093/nar/25.24.4876 9396791 PMC147148

[B11] Tamura K Peterson D Peterson N Stecher G Nei M Kumar S MEGA5: Molecular Evolutionary Genetics Analysis using Maximum Likelihood, Evolutionary Distance, and Maximum Parsimony Methods Mol Biol Evol 2011 10.1093/molbev/msr121 PMC3203626 21546353

[B12] Guindon S Dufayard JF Lefort V Anisimova M Hordijk W Gascuel O New algorithms and methods to estimate maximum-likelihood phylogenies: assessing the performance of PhyML 3.0 Syst Biol 2010 59 307 321 10.1093/sysbio/syq010 20525638

[B13] Anisimova M Gascuel O Approximate likelihood-ratio test for branches: A fast, accurate, and powerful alternative Syst Biol 2006 55 539 552 10.1080/10635150600755453 16785212

[B14] Li MH Fu SH Chen WX Wang HY Guo YH Genotype V Japanese Encephalitis Virus Is Emerging PLoS Negl Trop Dis 2011 5 7 e1231 10.1371/journal.pntd.0001231 21750744 PMC3130007

[B15] Mohammed MA Galbraith SE Radford AD Dove W Takasaki T Kurane I Solomon T Molecular phylogenetic and evolutionary analyses of Muar strain of Japanese encephalitis virus reveal it is the missing fifth genotype Infect Genet Evol 2011 11 855 862 10.1016/j.meegid.2011.01.020 21352956

[B16] Ni H Barrett AD Attenuation of Japanese encephalitis virus by selection of its mouse brain membrane receptor preparation escape variants Virology 1998 241 30 36 10.1006/viro.1997.8956 9454714

